# Comprehensive Learning-Enhanced Educational Competition Optimizer for Numerical Optimization and Reservoir Production Optimization

**DOI:** 10.3390/biomimetics11020111

**Published:** 2026-02-03

**Authors:** Shuaizhen Li, Jinxiong Luo

**Affiliations:** School of Earth Sciences, Yangtze University, Jingzhou 434023, China

**Keywords:** educational competition optimizer, comprehensive learning strategy, metaheuristic algorithm, global optimization, premature convergence, population diversity, benchmark functions

## Abstract

The performance of metaheuristic algorithms in solving high-dimensional, non-convex optimization problems is intricately linked to the balance between global exploration and local exploitation. Inspired by biomimetic principles of swarm intelligence, this study evaluates the Educational Competition Optimizer (ECO), a human learning-inspired metaheuristic, and addresses its vulnerability to rapid population homogenization and premature convergence in complex landscapes. To bridge the gap between rigid hierarchical competition and flexible biological cooperation, we propose the Comprehensive Learning-Enhanced Educational Competition Optimizer (CL-ECO), which introduces a dimension-wise multi-exemplar social learning mechanism to the ECO framework. Analogous to cooperative information sharing in animal swarms, CL-ECO reconstructs search trajectories by learning from different peers across decision variables, thereby promoting population diversity and adaptive exploration. Rigorous validation on the CEC 2017 benchmark suite demonstrates that CL-ECO achieves statistically superior convergence accuracy and robustness compared to seven state-of-the-art algorithms, securing the top Friedman rank (1.5862). Furthermore, the practical utility of CL-ECO is substantiated through a complex reservoir production optimization case study, where it outperforms the baseline algorithm in NPV maximization, proving its capability in managing complex, real-world engineering constraints.

## 1. Introduction

Optimization is fundamental to scientific inquiry and engineering design, serving as the cornerstone for maximizing efficiency and utility [1,2]. From calibrating high-fidelity computational models to orchestrating complex logistical networks [3,4], the objective remains constant: identifying the optimal configuration within a massive decision space. However, real-world problems often manifest as high-dimensional, non-convex landscapes riddled with local optima and non-linear interactions, rendering the global optimum elusive [5].

To address such complexity, a suite of traditional optimization methodologies has been historically developed [6]. Classical approaches, including gradient-based methods (e.g., steepest descent [7], Newton–Raphson [8]) and direct search algorithms, provide rigorous mathematical frameworks for seeking optimal solutions. These methods excel within well-defined, convex, and smooth problem domains where objective functions are continuous and differentiable, often guaranteeing convergence to a local optimum [9]. Their efficacy diminishes sharply, however, when confronted with the intricate realities of many contemporary problems. Pronounced limitations include a susceptibility to entrapment in local optima within non-convex landscapes, a reliance on gradient information that may be unavailable or computationally prohibitive, and an exponential escalation in computational cost with increasing dimensionality—a phenomenon known as the “curse of dimensionality” [10].

In response to these constraints, metaheuristic algorithms have emerged as a versatile and powerful class of optimization strategies [11,12,13]. Unlike traditional methods, metaheuristics operate according to higher-level guiding principles, enabling them to explore complex, non-convex, and high-dimensional search spaces without requiring gradient information. These strategies are commonly categorized into two principal lineages: evolutionary algorithms (EAs) and swarm intelligence (SI) algorithms. Evolutionary algorithms emulate processes of biological evolution; representative variants include the Genetic Algorithm (GA) [14] and Differential Evolution (DE) [15]. Conversely, swarm intelligence algorithms derive inspiration from collective intelligence in nature, with exemplary techniques such as Particle Swarm Optimization (PSO) [16,17] and Ant Colony Optimization (ACO) [18] mimicking social foraging behaviors. This inherent flexibility positions metaheuristics as a pivotal advancement, bridging the gap left by conventional approaches.

Among the real-world engineering challenges that demand such robust optimization capabilities, reservoir production optimization (RPO) stands out as a particularly critical high-dimensional problem. RPO aims to maximize the Net Present Value (NPV) of hydrocarbon assets by calibrating dynamic control settings. However, the underlying reservoir models are characterized by highly non-linear fluid dynamics, geological heterogeneity, and computationally expensive black-box simulations. These factors create a rugged, non-convex search landscape where traditional gradient-based methods struggle, making RPO an ideal testbed for advanced metaheuristic algorithms [19,20,21].

The proliferation of diverse metaheuristic paradigms raises a fundamental question: is there a single superior strategy? The No Free Lunch (NFL) theorem [22] provides a definitive answer, establishing that all optimization algorithms perform equally when averaged over all possible problems. This theoretical cornerstone suggests that any algorithmic gain in one domain is inevitably compensated by a performance deficit in another, thereby shifting the research focus from seeking a universal solver to the principled development of algorithms tailored for specific problem topologies. Driven by this imperative, the field has witnessed an explosion of innovative metaheuristics and hybridization strategies. Recent advancements include nature-inspired optimizers such as the Status-based Optimization (SBO) [23,24], Beaver Behavior Optimizer (BBO) [25], and the Comprehensive Learning Moss Growth Optimizer (CLMGO) [26], which demonstrate the efficacy of strategy integration in diverse domains. Other notable developments include the Parrot Optimizer (PO) [27], Escape behavior-based optimization [28], Moss Growth Optimization (MGO) [29], Slime Mould Algorithm (SMA) [30], and Colony Predation Algorithm (CPA) [31]. Furthermore, extensive research has focused on enhancing existing frameworks through multi-strategy integration, such as mutative crow search [32], forensic-based investigation enhancements [33], and horizontal–vertical crossover mechanisms [34]. These developments underscore a collective effort to balance exploration and exploitation more effectively within high-dimensional and non-convex landscapes.

Guided by this insight, this study focuses on the Educational Competition Optimizer (ECO) [35]. Recently proposed, ECO simulates optimization through a hierarchical progression mimicking primary, middle, and high school stages. Structurally, this simulates the natural evolution of foraging behaviors, transitioning from broad, dispersible exploration (primary stage) to focused, intensive resource exploitation (high school stage). Despite its innovative structure, ECO exhibits a notable structural vulnerability in information flow: typically, particles (students) learn predominantly from a single nearest leader or the global best. This centralized attraction mechanism, while efficient for simple unimodal landscapes, often precipitates a rapid decay in population diversity. Consequently, the algorithm becomes highly susceptible to premature convergence and stagnation in sub-optimal basins—a significant liability when navigating the rugged, high-dimensional landscapes of complex scenarios like Reservoir Production Optimization.

To address these limitations, this paper introduces the Comprehensive Learning-Enhanced Educational Competition Optimizer (CL-ECO). Drawing inspiration from biological mechanisms of social facilitation and cooperative foraging, the central innovation is the integration of an adaptive comprehensive learning (CL) strategy. This strategy functions as a “cooperative information sharing” module, akin to how animals in a swarm exchange information to locate food sources when individual searching fails. Whenever an individual is identified as stagnant, CL enables it to rebuild its search trajectory dimension-by-dimension, sampling information from the historical best positions of diverse exemplary peers rather than relying on a single guide. This multi-exemplar approach constructs a hybrid candidate solution that injects vital diversity into the population and provides a robust mathematical escape route from local optima. The principal contributions of this work are threefold:

- Algorithmic Innovation: We introduce an algorithmically tailored enhancement designed to mitigate the premature convergence and diversity loss of the canonical ECO. This mechanism facilitates multi-source information exchange, fundamentally enhancing global exploration capability.
- Benchmark Validation: We formulate the CL-ECO algorithm and conduct a rigorous evaluation against state-of-the-art metaheuristics on the CEC 2017 benchmark suite. Statistical tests, including Friedman and Wilcoxon signed-rank tests, confirm its significant competitive performance.
- Engineering Application: We validate the practical utility of CL-ECO by applying it to the challenging real-world problem of specialized reservoir production optimization. The results show that CL-ECO maximizes the Net Present Value (NPV) more effectively than competing algorithms, confirming its robustness in handling complex engineering constraints.

The remainder of this paper is organized as follows: Section 2 outlines the canonical ECO. Section 3 details the proposed CL-ECO and the comprehensive learning mechanism. Section 4 presents the experimental analysis on benchmark functions. Section 5 discusses the application to reservoir engineering. Finally, Section 6 concludes the study.

## 2. Original Educational Competition Optimizer

The Educational Competition Optimizer (ECO) [35] is a nature-inspired metaheuristic that abstracts the dynamics of an educational system to drive optimization. The algorithm enables a structured transition from exploration to exploitation by simulating the educational progression of students through primary, middle, and high school stages. The population is dynamically partitioned into “schools” (leaders) and “students” (followers) based on fitness rankings, with the intensity of competition increasing as the iterations proceed. The mathematical formulation of these stages is detailed below.

### 2.1. Mechanisms

1. Initialization via Chaotic Mapping:

ECO employs a logistic chaotic map to distribute the initial population. This technique promotes initial diversity by ensuring more uniform coverage of the search space:

(1) $$x_{i}=\alpha \cdot x_{i-1}\cdot \left(1-x_{i-1}\right),\;0\leq x_{0}\leq 1,\alpha =4,$$

(2) $$X_{i}=lb+\left(ub-lb\right)\cdot x_{i},$$

where $X_{i}$ represents the initial position of the *i*-th individual, while lb and ub denote the lower and upper bounds of the decision variables, respectively.

2. Primary School Stage (Exploration):

In the early phase, the algorithm prioritizes global exploration. The top 20% of the population are designated as schools. Schools update their positions by drifting towards the population mean to consolidate the search, while students perform a Levy flight-based random walk towards their nearest school:

(3) $$X_{i}^{t+1}=X_{i}^{t}+w\cdot \left(X_{i,mean}^{t}-X_{i}^{t}\right)\cdot Levy\left(dim\right),$$

(4) $$X_{i}^{t+1}=X_{i}^{t}+w\cdot \left(close\left(X_{i}^{t}\right)-X_{i}^{t}\right)\cdot randn.$$

Equation (3) utilizes the population mean to promote group cohesion during exploration, while the Levy flight component in Equation (4) introduces high-variance random perturbations, allowing the schools to “jump” across local optima. The use of Gaussian random walks in Equation (5) enables students to fine-tune their search around the vicinity of their assigned leaders. Here, w=0.1ln(2−t/Max_iter) is a time-varying weight, $X_{i,mean}^{t}$ is the mean position, and $\mathrm{close}(X_{i}^{t})$ is the nearest school. This stage fosters wide-ranging exploration of the optimization landscape.

3. Middle School Stage (Transition):

As the search progresses, the focus shifts towards a balance of exploration and exploitation. The school ratio tightens to 10%. Schools begin to incorporate information from the global best solution ($X_{best}^{t}$), while students are segregated based on a “talent” mechanism that probabilistically adjusts their learning strategy:

(5) $$X_{i}^{t+1}=X_{i}^{t}+\left(X_{best}^{t}-X_{mean}^{t}\right)\cdot \exp\left(\frac{t}{\mathrm{Max}\_\mathrm{iter}}-1\right)\cdot Levy\left(dim\right),$$

(6) $$X_{i}^{t+1}=\left\{\begin{array}{cc} X_{i}^{t}-w\cdot close\left(X_{i}^{t}\right)-P\cdot \left(E\cdot w\cdot close\left(X_{i}^{t}\right)-X_{i}^{t}\right), & \mathrm{if}\;R_{1}<H\\ X_{i}^{t}-w\cdot close\left(X_{i}^{t}\right)-P\cdot \left(w\cdot close\left(X_{i}^{t}\right)-X_{i}^{t}\right), & \mathrm{if}\;R_{1}\geq H \end{array}\right.,$$

In this transitional phase, Equation (6) draws schools towards the global best to increase selection pressure. Equation (7) introduces “patience” (*P*) and “motivation” (*E*) factors to simulate stochastic student behavior, balancing the focus between global attraction and individual experience. where *E* and *P* are motivation and patience factors, respectively, introducing stochastic perturbations to prevent stagnation.

4. High School Stage (Exploitation):

In the final phase, the algorithm focuses on fine-tuning the best solutions. Schools interact with both the best and worst individuals to refine their positions, while all students converge directly towards the global best, maximizing selection pressure:

(7) $$X_{i}^{t+1}=X_{i}^{t}+\left(X_{best}^{t}-X_{i}^{t}\right)\cdot randn-\left(X_{worst}^{t}-X_{i}^{t}\right)\cdot randn,$$

(8) $$X_{i}^{t+1}=\left\{\begin{array}{cc} X_{b}est^{t}-P\cdot \left(E\cdot X_{best}^{t}-X_{i}^{t}\right), & \mathrm{if}\;R_{2}<H\\ X_{b}est^{t}-P\cdot \left(X_{best}^{t}-X_{i}^{t}\right), & \mathrm{if}\;R_{2}\geq H \end{array}\right..$$

Finally, Equation (8) enables schools to refine their positions by interacting with the population extremes, while Equation (9) forces rapid convergence of all students toward the global best for precise local refinement. This stage ensures efficient convergence toward the global optimum.

5. Greedy Selection:

Following each update operation, a greedy selection mechanism is applied. The new position $X_{i}^{t+1}$ replaces $X_{i}^{t}$ if and only if it yields a superior fitness value, ensuring monotonic improvement of the objective function.

### 2.2. Integrated Iteration

**Figure 1:**
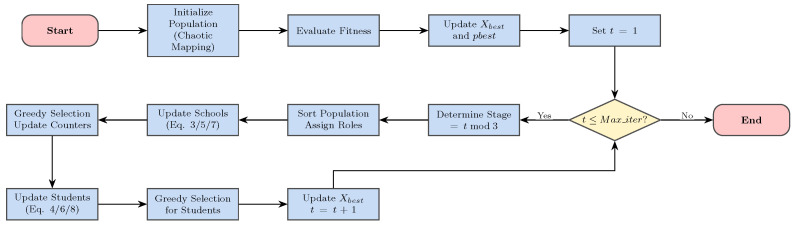
Flowchart of the original ECO algorithm.

The original ECO algorithm integrates these stages into a cyclic process. The population status is updated iteratively, with the stage determined by the modulus of the iteration count. This structure provides a rudimentary balance between search phases but remains vulnerable to diversity collapse in high-dimensional spaces. The complete workflow is depicted in Figure 1.

## 3. Proposed CL-ECO Algorithm

### 3.1. Comprehensive Learning Strategy

The primary limitation of the original ECO is its reliance on a strict hierarchy where students learn predominantly from the nearest leader or the global best. While efficient for unimodal problems, this tight coupling often leads to rapid diversity loss in multimodal landscapes. To mitigate this, we introduce a comprehensive learning (CL) strategy. Inspired by the CL-PSO paradigm, this mechanism effectively decouples the search dimensions, allowing an individual to construct a “hybrid candidate” by learning variable-by-variable from different peers.

The CL strategy operates through four integrated components:

- Stagnation Monitor: A stagnation counter is maintained for each individual. If the personal best (pbest) fails to update for a specified number of consecutive generations (stagnation_limit), the CL strategy is triggered. This conditional activation ensures computational resources are allocated only to stagnant individuals.
- Rank-Based Learning Probability: A learning probability $P_{c_{i}}$ is assigned to each individual *i* based on its fitness rank. Lower-ranked individuals are assigned higher learning probabilities to facilitate larger perturbations, while higher-ranked individuals retain more personal information:
  (9) $$P_{c_{i}}=a+b\times \frac{\exp\left(10\cdot \frac{i-1}{N-1}\right)-1}{\exp(10)-1},$$
  Equation (10) ensures that individuals with low fitness ranks (higher indices) are assigned higher $P_{c_{i}}$ values, encouraging them to learn more from varied exemplary peers rather than their own histories to facilitate escape from local optima. where *i* is the rank index (1 being the best), and a=0.05,b=0.45 are empirical constants.
- Dimension-Wise Exemplar Sampling: For each dimension *j* of a stagnant individual *i*, the algorithm determines its learning source based on the probability $P_{c_{i}}$. If a randomly generated number surpasses $P_{c_{i}}$, the dimension *j* learns from its own experience ($pbest_{i}$). Otherwise, a tournament selection process is invoked: two random peers are selected from the population, and the one with the superior fitness value is chosen as the exemplar for that specific dimension. This mechanism constructs a composite guide vector ${\hat{X}}_{i}$ that aggregates diverse information from across the population:
  (10) $$f_{i}\left(j\right)=\left\{\begin{array}{cc} \mathrm{Tournament}(k_{1},k_{2}), & \mathrm{if}\;\mathrm{rand}<P_{c_{i}}\\ i, & \mathrm{otherwise} \end{array}\right.,$$
  This dimension-wise sampling allows the algorithm to decompose a high-dimensional problem into multiple independent variable searches, aggregation of which yields a “hybrid” guide vector that represents an unconventional search direction.
- Constructive Position Update: The new candidate position is generated by learning from the composite exemplar:
  (11) $$X_{i}^{new}\left(j\right)=X_{i}\left(j\right)+r\cdot \left({\mathrm{pbest}}_{f_{i}\left(j\right)}\left(j\right)-X_{i}\left(j\right)\right).$$
  To prevent the degenerate case where an individual learns entirely from itself, a safeguard ensures that at least one dimension is forced to learn from another particle if $f_{i}\left(j\right)=i$ for all *j*.

It is important to distinguish the proposed CL strategy from the standard Comprehensive Learning Particle Swarm Optimization (CL-PSO). While we adopt the dimension-wise multi-exemplar concept, our mechanism is integrated into the hierarchical ECO framework as a conditional “remedial” operator. Unlike CL-PSO, which utilizes comprehensive learning as the primary search strategy throughout the run, CL-ECO triggers the CL update only when an individual is identified as stagnant. This selective activation preserves the rapid convergence efficiency of the canonical ECO while providing a robust escape route from local optima only when necessary. Furthermore, the interaction between the comprehensive learning guide vector and the ECO’s stage-specific roles creates a hybrid search dynamics tailored for complex landscapes.

### 3.2. The CL-ECO Framework

The proposed CL-ECO integrates this strategy as a “remedial” operator. In each iteration, standard ECO dynamics drive the primary search. However, individuals identified as stagnant enter the comprehensive learning phase. Crucially, the algorithm generates a candidate solution via the standard ECO update and the CL update (if triggered), retaining the one that yields the superior fitness improvement.

**Figure 2:**
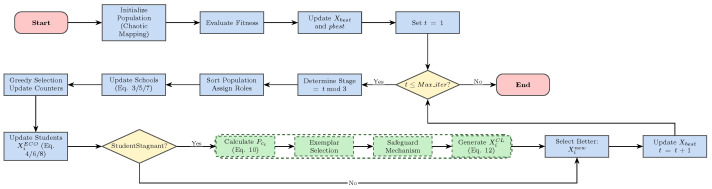
Flowchart of the proposed CL-ECO, highlighting the conditional stagnation–response mechanism.

This dual-track mechanism preserves the fast convergence characteristics of the original ECO while injecting targeted diversity through orthogonal learning when entrapment occurs. The flowchart in Figure 2 illustrates this extensive decision process.

Algorithm 1 outlines the complete pseudocode.
**Algorithm 1** Pseudocode of CL-ECO.1:**Initialize** population *X*, velocities, and parameters.2:Evaluate initial fitness; initialize pbest and gbest.3:**while** t≤Max_iter **do**4:    Determine competition stage (Primary/Middle/High).5:    Sort population and assign roles (Schools/Students).6:    **for** each individual i=1 to *N* **do**7:        **Standard Update:** Generate candidate $X_{i}^{ECO}$ using ECO rules.8:        **if** Student *i* is **stagnant then**9:           Calculate $P_{c_{i}}$.10:           **for** j=1 to dim **do**11:               Select exemplar index $f_{i}\left(j\right)$ via tournament logic.12:           **end for**13:           **Safeguard:** Ensure ∃j, $f_{i}\left(j\right)\neq i$.14:           **CL Update:** Generate candidate $X_{i}^{CL}$ using composite exemplar.15:           Select better candidate: $X_{i}^{new}=\arg\min\left(f\left(X_{i}^{ECO}\right),f\left(X_{i}^{CL}\right)\right)$.16:        **else**17:           $X_{i}^{new}=X_{i}^{ECO}$.18:        **end if**19:        **Evaluation:** Evaluate $f(X_{i}^{new})$.20:        **Selection:** If $f\left(X_{i}^{new}\right)<pfit_{i}$, update $X_{i}$ and $pbest_{i}$; reset stagnation counter. Else, increment counter.21:    **end for**22:    Update global best $X_{best}$.23:    t=t+1.24:**end while**

The time complexity of CL-ECO is dictated by the population size *N*, dimension *D*, and iterations *T*. The base ECO operations (sorting, updating) require O(NlogN+N·D). The CL strategy adds a conditional overhead of O(N·D) in the worst case (all stagnant). Thus, the total complexity O(T(NlogN+N·D)) is asymptotically equivalent to that of the canonical ECO, ensuring that the performance gains do NOT come at the cost of prohibitive computational expense.

## 4. Experimental Results and Discussion

This section presents a rigorous empirical validation of the proposed CL-ECO framework. To ensure reproducibility and statistical validity, all experiments were performed on a standardized computational platform (MATLAB R2024a, Intel Core 13700KF, Intel, Santa Clara, CA, USA, 16 GB RAM) under strict control parameters. The population size was fixed at N=50 with a computational budget of MaxFEs = 300,000. Each algorithm was executed for 30 independent runs to mitigate stochastic variance. Performance is quantified using the mean (Avg) and standard deviation (Std) of the objective function values.

### 4.1. Benchmark Test Suite

The evaluation utilizes the CEC 2017 benchmark suite [36,37], a widely recognized collection of 29 scalable optimization problems categorized into three groups: unimodal functions (F1, F3), characterized by a single global optimum to test convergence velocity and exploitation precision; multimodal functions (F4–F10), possessing exponentially many local optima to rigorously challenge global exploration and stagnation avoidance capabilities; and hybrid (F11–F20) as well as composition (F21–F30) functions, which simulate real-world complexity by superimposing diverse sub-landscapes to assess the algorithm’s versatility and robustness. Table 1 details the specific properties of these functions.

### 4.2. Comparative Performance Analysis

The efficacy of CL-ECO is benchmarked against the original ECO [35] and six leading metaheuristics: Moss Growth Optimization (MGO) [29], Parrot Optimizer (PO) [27], Beaver Behavior Optimizer (BBO) [25], Differential Evolution (DE) [15], Hunger Games Search (HGS) [38], and Colony Predation Algorithm (CPA) [31]. Identical initialization and termination criteria were applied to ensure a fair comparison. The results, summarized in Table 2 and Table 3, elucidate the performance hierarchy. As evidenced by Table 2, CL-ECO demonstrates a commanding performance, securing the top position with a Friedman mean rank of 1.5862. This ranking significantly outstrips the original ECO (3.3103) and the closest competitor, DE (4.0). Regarding the algorithmic configuration, the key hyper-parameters of CL-ECO, including the stagnation limit and the learning probability constants (a=0.05,b=0.45), are adopted from the recently proposed CLMGO framework [26], as these settings have shown robust performance in complex engineering optimization. Utilizing these literature-verified values ensures a stable and reproducible baseline for our comparative analysis.

This consistent performance suggests that the CL strategy does not merely offer sporadic improvements but fundamentally enhances the algorithm’s consistency across diverse problem structures. Complementing this ranking, the Wilcoxon signed-rank test results (Table 3) provide statistical confirmation of this superiority. Against algorithms like MGO and PO, CL-ECO frequently achieves machine-level precision differences ($p\approx 1.73\times 10^{-6}$), underscoring its robustness.

Regarding landscape adaptability, CL-ECO exhibits versatile performance across different problem classes. On unimodal functions (F1, F3), it matches or exceeds the precision of ECO, validating that the integration of the CL strategy does not compromise local convergence speed. Conversely, on complex multimodal (F6, F9) and hybrid functions (F11–F20), CL-ECO consistently locates superior basins of attraction. For instance, on F6, CL-ECO reduces the mean error by approximately 5% compared to ECO. This confirms that the dimension-wise learning mechanism successfully diversifies the search trajectory, effectively mitigating the prematurity observed in the canonical algorithm.

**Figure 3:**
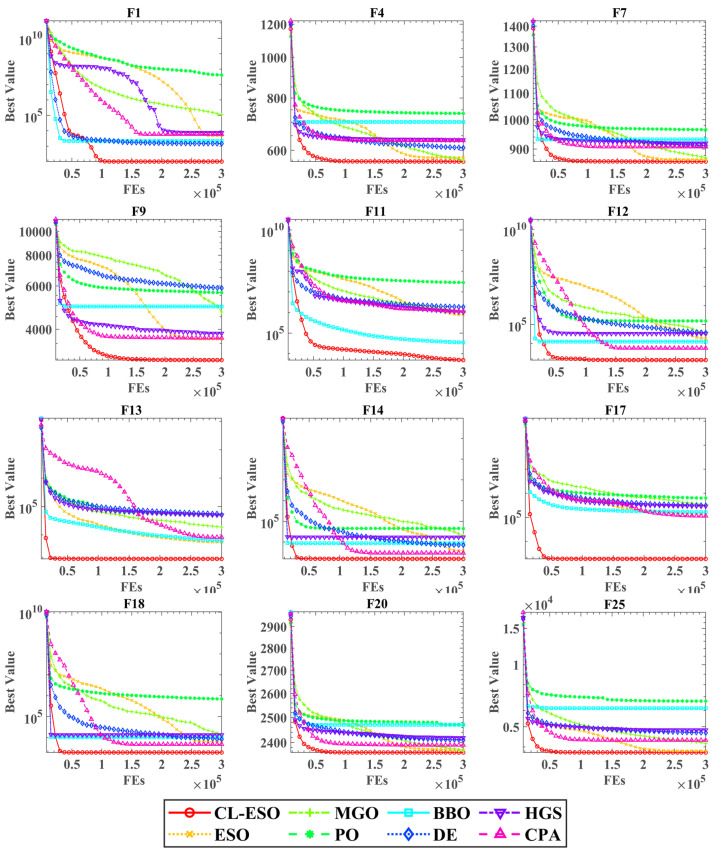
Convergence behavior analysis on nine representative benchmark functions.

The convergence profiles in Figure 3 visually reinforce these findings. CL-ECO (red trajectory) exhibits a characteristic “rapid-descent” behavior in the early phases, indicative of efficient exploration, generally followed by a deeper convergence plateau. This validates the hypothesis that the CL strategy acts as an effective “stagnation trigger,” reactivating the search when standard operators falter.

However, it is observable that the performance improvement of CL-ECO over ECO is more significant in complex multimodal and hybrid functions than in simpler unimodal landscapes like F2. In relatively smooth or single-basin domains, the original ECO hierarchy is already highly optimized, and the trigger frequency of the CL strategy remains minimal. This explains the marginal nature of performance gains in such instances, contrasting with the substantial breakthroughs achieved in rugged, high-dimensional search spaces where the canonical algorithm frequently encounters stagnation checkpoints.

## 5. Application to Production Optimization

Reservoir production optimization (RPO) represents a fundamental high-dimensional engineering challenge, critical for maximizing the economic lifecycle of hydrocarbon assets. The objective is to determine the optimal control trajectory, which encompasses water injection rates and bottom-hole pressures (BHPs) to maximize the Net Present Value (NPV). This problem is mathematically intractable for classical methods due to the highly non-linear fluid dynamics governing subsurface flow (e.g., multiphase Darcy flow) and the non-convex nature of the objective function. Consequently, it serves as an ideal testbed for assessing the practical efficacy of advanced metaheuristics.

To evaluate the efficacy of the proposed algorithm in a practical setting, a simulation-based framework is adopted. The industry-standard Eclipse reservoir simulator acts as the “black-box” forward model to evaluate candidate production schedules, calculating dynamic reservoir responses to the specified controls [39,40,41]. In a representative application, the improved algorithm is tasked with optimizing the injection and production strategies for a multi-well reservoir model over several control steps, with the simulator providing the necessary production data (oil, water, and gas rates) for each proposed schedule.

The performance of each control strategy is evaluated using the Net Present Value (NPV), which aggregates discounted future cash flows. The objective function to be maximized is formally defined as

(12) $$NPV\left(u\right)=\sum_{t=1}^{N_{t}}\frac{\Delta t_{t}\cdot \left(r_{o}Q_{o,t}-r_{w}Q_{w,t}-r_{i}Q_{i,t}\right)}{{\left(1+b\right)}^{p_{t}}},$$

where u denotes the control vector. The specific economic parameters are: oil price $r_{o}$,water handling costs $r_{w},r_{i}$, and discount rate *b*. Non-linear production constraints, such as minimum bottom-hole pressure (BHP) limits for producers and maximum water-cut thresholds, are omitted in this specific study to isolate and evaluate the core algorithmic search capability. Future higher-fidelity studies will integrate these operational constraints to further assess the algorithm’s industrial applicability.

### 5.1. Reservoir Model Description

**Figure 4:**
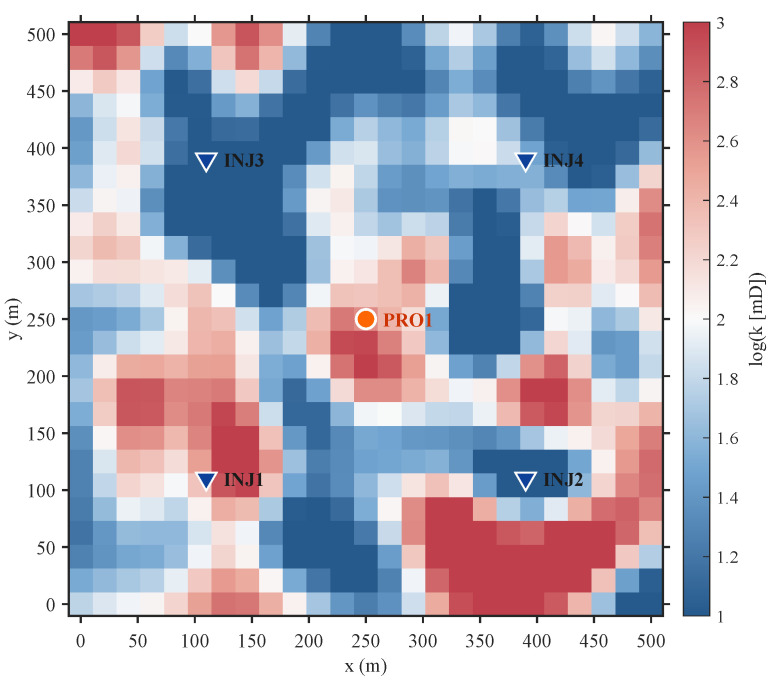
Two-dimensional synthetic reservoir model and well pattern arrangement.

A two-dimensional, heterogeneous synthetic reservoir model is developed to simulate the intricate architecture of a fluvial channel system. It adopts a classical inverted five-spot well arrangement, with one central producer (PRO1) and four injectors (INJ1–INJ4) at the periphery, illustrated in Figure 4. This design facilitates a robust assessment of the optimization methodology under geologically realistic, spatially variable conditions.

The model domain is spatially discretized using a 25×25 Cartesian grid, resulting in 625 active cells. Each cell possesses a uniform thickness and planar dimensions of 30m×30m. Porosity is held constant at a value of 0.2. In contrast, the permeability field is stochastically generated to embed high-permeability channels amidst low-permeability background regions, creating preferential flow pathways. The resulting log-permeability distribution, ln(K), which dictates fluid flow behavior, is presented in Figure 4.

The optimization framework is configured with flexibility. The total simulation duration is set to 1000 days, partitioned into 12 control steps. Optimization involves five wells, yielding a problem with 60 decision variables. The goal is to maximize the Net Present Value (NPV) by determining optimal well controls. Economic parameters are assigned plausible values: oil price (85 USD/STB), water injection cost (5 USD/STB), water processing cost (3 USD/STB), and an annual discount rate (3%). These settings maintain realism while concentrating the analysis on the core optimization algorithm’s performance.

### 5.2. Results and Discussion

**Figure 5:**
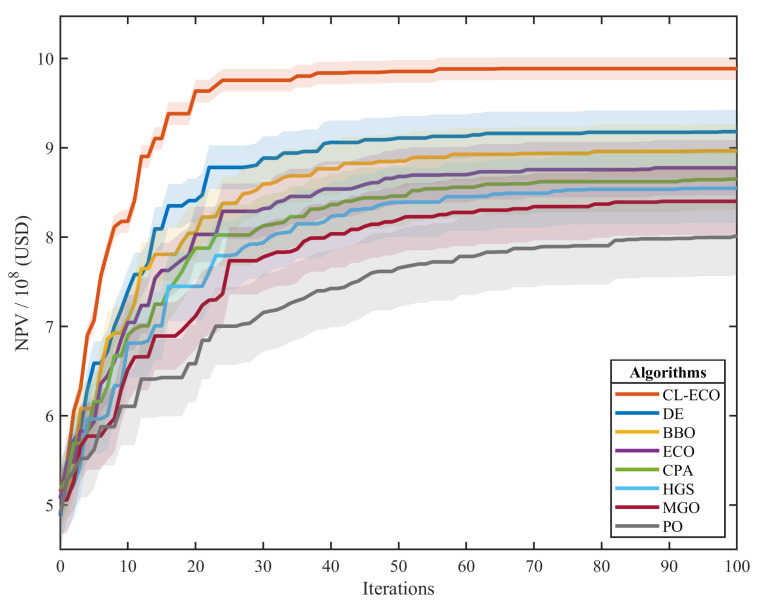
Convergence curve analysis of all algorithms on the production optimization problem (the shaded area represents the standard deviation).

The performance of CL-ECO is benchmarked against the original ECO and the competitor algorithms under identical conditions (five independent runs). Table 4 summarizes the statistical outcomes. Regarding economic performance, CL-ECO achieves a mean NPV of $9.842 × 10^8^ , surpassing the baseline ECO ($8.745 × 10^8^) by a margin of 12.5%. The distinct advantage of CL-ECO in this reservoir model stems from its dimension-wise learning capability. In geologically heterogeneous fields where fluvial channels create preferential but isolated flow paths, standard exploitative algorithms like ECO tend to lock onto a single moderate-performing injection scheme across all wells. By allowing the control steps of different wells to be optimized independently through learning from diverse exemplars, CL-ECO maintains the flexibility required to discover alternative, better sweep patterns across the model. This significant financial advantage stems from the algorithm’s enhanced ability to navigate the complex trade-offs between injection support and production efficiency within the heterogeneous permeability field. By identifying high-transmissibility pathways more effectively, CL-ECO optimizes the sweep efficiency, thereby recovering more oil at lower water-cut levels. In terms of operational stability, the standard deviation of CL-ECO remains the lowest among all evaluated algorithms ($1.27 × 10^7^), suggesting that its “CL-driven search reconstruction” mechanism consistently identifies high-quality basins regardless of initial stochastic seeding. In sharp contrast, competitors like Parrot Optimizer (PO) exhibit large variances ($4.33 × 10^7^), indicating a high dependency on initial conditions and a tendency for unstable convergence. Furthermore, as illustrated by the convergence trajectories in Figure 5, CL-ECO (red) identifies superior solutions significantly earlier than its counterparts. The rapid early-stage ascent reflects its high exploration efficiency, confirmed by the algorithm’s ability to quickly bypass local optima traps that frequently hinder the canonical ECO and MGO.

In summary, the integration of dimension-wise comprehensive learning grants CL-ECO a dual advantage: it not only maximizes absolute economic returns but also ensures a robust, repeatable decision-making process. These characteristics are critical for field-scale reservoir management, where stable and high-performance well controls are paramount.

## 6. Conclusions

This study introduced the Comprehensive Learning-Enhanced Educational Competition Optimizer (CL-ECO), a targeted advancement of the canonical ECO framework designed to address critical limitations in population diversity and its susceptibility to local optima in complex optimization tasks. This is realized through the novel integration of a dimension-wise, multi-exemplar comprehensive learning (CL) strategy, which enables stagnant individuals to reconstruct their search trajectories by sampling information from diverse peers. The resulting framework effectively decouples decision variables, fostering a robust balance between global exploration and local exploitation while preventing the premature stagnation inherent in the original hierarchical structure.

The efficacy of the proposed CL-ECO was substantiated through rigorous empirical validation. In comprehensive assessments on the CEC 2017 benchmark suite, CL-ECO demonstrated superior performance, securing the premier rank in Friedman statistical tests with a score of 1.5862 and showing a statistically validated advantage over established metaheuristics across unimodal, multimodal, and hybrid landscapes. Furthermore, its practical utility was confirmed in a high-fidelity reservoir production optimization case study. In this application, CL-ECO delivered a mean Net Present Value (NPV) of $9.842 × 10^8^, representing a 12.5% improvement over the baseline algorithm with exceptional consistency, thereby validating its reliability for handling complex, real-world engineering constraints. Despite these advancements, the effectiveness of the CL-ECO framework remains contingent upon the proper calibration of the stagnation_limit. An overly aggressive trigger may introduce excessive diversity and computational overhead (O(N·D) per stagnant individual), while a delayed response might hinder the timely escape from local optima. Nevertheless, the algorithm’s dimension-wise reconstruction capability effectively decouples decision variables, ensuring robustness in rugged, non-separable landscapes. This characteristic makes CL-ECO highly adaptable to a broad spectrum of engineering applications, including those involving stochastic or fuzzy uncertainties. Building on these promising results, future research will pursue three key trajectories: (1) conducting systematic ablation studies to quantify the contribution of the stagnation trigger; (2) developing adaptive parameter control mechanisms and extensions for high-dimensional and multi-objective domains; and (3) exploring broader industrial challenges, such as multi-well placement and automatic history matching, to further solidify its status as a robust optimization tool.

## Figures and Tables

**Table 1:** CEC2017 benchmark functions.

Function	Function Name	Class	Optimum
F1	Shifted and Rotated Bent Cigar Function	Unimodal	100
F3	Shifted and Rotated Zakharov Function	Unimodal	300
F4	Shifted and Rotated Rosenbrock Function	Multimodal	400
F5	Shifted and Rotated Rastrigin Function	Multimodal	500
F6	Shifted and Rotated Expanded Scaffer F6 Function	Multimodal	600
F7	Shifted and Rotated Lunacek Bi-Rastrigin Function	Multimodal	700
F8	Shifted and Rotated Non-Continuous Rastrigin Function	Multimodal	800
F9	Shifted and Rotated Lévy Function	Multimodal	900
F10	Shifted and Rotated Schwefel Function	Multimodal	1000
F11	Hybrid Function 1 (N = 3)	Hybrid	1100
F12	Hybrid Function 2 (N = 3)	Hybrid	1200
F13	Hybrid Function 3 (N = 3)	Hybrid	1300
F14	Hybrid Function 4 (N = 4)	Hybrid	1400
F15	Hybrid Function 5 (N = 4)	Hybrid	1500
F16	Hybrid Function 6 (N = 4)	Hybrid	1600
F17	Hybrid Function 6 (N = 5)	Hybrid	1700
F18	Hybrid Function 6 (N = 5)	Hybrid	1800
F19	Hybrid Function 6 (N = 5)	Hybrid	1900
F20	Hybrid Function 6 (N = 6)	Hybrid	2000
F21	Composition Function 1 (N = 3)	Composition	2100
F22	Composition Function 2 (N = 3)	Composition	2200
F23	Composition Function 3 (N = 4)	Composition	2300
F24	Composition Function 4 (N = 4)	Composition	2400
F25	Composition Function 5 (N = 5)	Composition	2500
F26	Composition Function 6 (N = 5)	Composition	2600
F27	Composition Function 7 (N = 6)	Composition	2700
F28	Composition Function 8 (N = 6)	Composition	2800
F29	Composition Function 9 (N = 3)	Composition	2900
F30	Composition Function 10 (N = 3)	Composition	3000

**Table 2:** Results of the CL-ECO and other algorithms on CEC2017 benchmark functions.

	F1		F2		F3	
**Algo.**	**Avg**	**Std**	**Avg**	**Std**	**Avg**	**Std**
CL-ECO	$1.0000\times 10^{2}$	$3.1995\times 10^{-14}$	$3.0334\times 10^{2}$	$1.1726\times 10^{1}$	$4.1204\times 10^{2}$	$2.2779\times 10^{1}$
ECO	$4.7374\times 10^{3}$	$5.0546\times 10^{3}$	$3.0000\times 10^{2}$	$2.6608\times 10^{-3}$	$4.8211\times 10^{2}$	$3.5284\times 10^{1}$
MGO	$1.1042\times 10^{5}$	$7.9604\times 10^{4}$	$4.5469\times 10^{4}$	$1.2909\times 10^{4}$	$4.9482\times 10^{2}$	$1.4155\times 10^{1}$
PO	$4.1186\times 10^{7}$	$5.2998\times 10^{7}$	$4.8755\times 10^{3}$	$2.9181\times 10^{3}$	$5.1333\times 10^{2}$	$2.0310\times 10^{1}$
BBO	$2.1583\times 10^{3}$	$2.1367\times 10^{3}$	$3.0000\times 10^{2}$	$4.7556\times 10^{-3}$	$4.6312\times 10^{2}$	$2.6713\times 10^{1}$
DE	$1.4582\times 10^{3}$	$2.6050\times 10^{3}$	$1.9849\times 10^{4}$	$3.4189\times 10^{3}$	$4.9077\times 10^{2}$	$8.9895\times 10^{0}$
HGS	$7.4728\times 10^{3}$	$6.1449\times 10^{3}$	$2.2896\times 10^{3}$	$4.5148\times 10^{3}$	$4.9149\times 10^{2}$	$2.6808\times 10^{1}$
CPA	$5.9278\times 10^{3}$	$6.4034\times 10^{3}$	$3.0000\times 10^{2}$	$1.7306\times 10^{-7}$	$4.7970\times 10^{2}$	$3.1429\times 10^{1}$
	**F4**		**F5**		**F6**	
**Algo.**	**Avg**	**Std**	**Avg**	**Std**	**Avg**	**Std**
CL-ECO	$5.6398\times 10^{2}$	$1.2454\times 10^{1}$	$6.0000\times 10^{2}$	$1.8342\times 10^{-4}$	$7.8961\times 10^{2}$	$1.1755\times 10^{1}$
ECO	$5.7645\times 10^{2}$	$1.8764\times 10^{1}$	$6.0113\times 10^{2}$	$1.8370\times 10^{0}$	$8.2966\times 10^{2}$	$2.7009\times 10^{1}$
MGO	$5.6832\times 10^{2}$	$1.3372\times 10^{1}$	$6.0000\times 10^{2}$	$1.7944\times 10^{-4}$	$7.9939\times 10^{2}$	$1.4397\times 10^{1}$
PO	$7.3498\times 10^{2}$	$4.5435\times 10^{1}$	$6.5755\times 10^{2}$	$7.9024\times 10^{0}$	$1.1420\times 10^{3}$	$7.0276\times 10^{1}$
BBO	$7.0135\times 10^{2}$	$3.8391\times 10^{1}$	$6.4660\times 10^{2}$	$6.7789\times 10^{0}$	$1.0016\times 10^{3}$	$7.0418\times 10^{1}$
DE	$6.0865\times 10^{2}$	$7.8313\times 10^{0}$	$6.0000\times 10^{2}$	$0.0000\times 10^{0}$	$8.4613\times 10^{2}$	$7.9328\times 10^{0}$
HGS	$6.3513\times 10^{2}$	$2.8001\times 10^{1}$	$6.0133\times 10^{2}$	$2.8043\times 10^{0}$	$8.9213\times 10^{2}$	$3.9842\times 10^{1}$
CPA	$6.3348\times 10^{2}$	$3.1826\times 10^{1}$	$6.0000\times 10^{2}$	$4.1274\times 10^{-4}$	$8.4722\times 10^{2}$	$2.8126\times 10^{1}$
	**F7**		**F8**		**F9**	
**Algo.**	**Avg**	**Std**	**Avg**	**Std**	**Avg**	**Std**
CL-ECO	$8.6021\times 10^{2}$	$9.2229\times 10^{0}$	$9.7436\times 10^{2}$	$4.9570\times 10^{1}$	$2.9959\times 10^{3}$	$2.8415\times 10^{2}$
ECO	$8.6656\times 10^{2}$	$1.7558\times 10^{1}$	$1.1645\times 10^{3}$	$2.8758\times 10^{2}$	$3.6488\times 10^{3}$	$5.5086\times 10^{2}$
MGO	$8.7162\times 10^{2}$	$1.6127\times 10^{1}$	$9.4124\times 10^{2}$	$3.6740\times 10^{1}$	$4.7039\times 10^{3}$	$3.7970\times 10^{2}$
PO	$9.6496\times 10^{2}$	$2.6158\times 10^{1}$	$4.8087\times 10^{3}$	$7.9508\times 10^{2}$	$5.6408\times 10^{3}$	$6.5322\times 10^{2}$
BBO	$9.3173\times 10^{2}$	$2.3627\times 10^{1}$	$4.2090\times 10^{3}$	$1.1110\times 10^{3}$	$4.9491\times 10^{3}$	$6.6775\times 10^{2}$
DE	$9.1124\times 10^{2}$	$9.0949\times 10^{0}$	$9.0000\times 10^{2}$	$6.3333\times 10^{-14}$	$5.8831\times 10^{3}$	$2.9749\times 10^{2}$
HGS	$9.1884\times 10^{2}$	$2.3158\times 10^{1}$	$3.8897\times 10^{3}$	$1.0354\times 10^{3}$	$3.8480\times 10^{3}$	$5.2132\times 10^{2}$
CPA	$9.0735\times 10^{2}$	$1.7759\times 10^{1}$	$2.2485\times 10^{3}$	$5.1410\times 10^{2}$	$3.6888\times 10^{3}$	$4.8776\times 10^{2}$
	**F10**		**F11**		**F12**	
**Algo.**	**Avg**	**Std**	**Avg**	**Std**	**Avg**	**Std**
CL-ECO	$1.1248\times 10^{3}$	$1.2796\times 10^{1}$	$4.8702\times 10^{3}$	$7.4937\times 10^{3}$	$1.3407\times 10^{3}$	$1.0970\times 10^{1}$
ECO	$1.2208\times 10^{3}$	$4.0077\times 10^{1}$	$7.7321\times 10^{5}$	$5.6407\times 10^{5}$	$1.8818\times 10^{4}$	$9.6181\times 10^{3}$
MGO	$1.1898\times 10^{3}$	$2.1227\times 10^{1}$	$9.7482\times 10^{5}$	$5.4662\times 10^{5}$	$2.8560\times 10^{4}$	$1.7966\times 10^{4}$
PO	$1.3074\times 10^{3}$	$8.4786\times 10^{1}$	$2.8135\times 10^{7}$	$2.4764\times 10^{7}$	$1.5334\times 10^{5}$	$1.2857\times 10^{5}$
BBO	$1.2192\times 10^{3}$	$2.9512\times 10^{1}$	$3.5033\times 10^{4}$	$1.3690\times 10^{4}$	$1.2641\times 10^{4}$	$1.6151\times 10^{4}$
DE	$1.1669\times 10^{3}$	$2.4350\times 10^{1}$	$1.8414\times 10^{6}$	$1.3372\times 10^{6}$	$3.7213\times 10^{4}$	$1.8881\times 10^{4}$
HGS	$1.2055\times 10^{3}$	$3.1263\times 10^{1}$	$1.0242\times 10^{6}$	$7.3489\times 10^{5}$	$3.3017\times 10^{4}$	$2.6217\times 10^{4}$
CPA	$1.1862\times 10^{3}$	$3.6782\times 10^{1}$	$1.1743\times 10^{6}$	$8.3936\times 10^{5}$	$5.7802\times 10^{3}$	$5.7431\times 10^{3}$
	**F13**		**F14**		**F15**	
**Algo.**	**Avg**	**Std**	**Avg**	**Std**	**Avg**	**Std**
CL-ECO	$1.4333\times 10^{3}$	$1.3175\times 10^{1}$	$1.5181\times 10^{3}$	$8.6553\times 10^{0}$	$2.2159\times 10^{3}$	$1.8480\times 10^{2}$
ECO	$5.5636\times 10^{3}$	$4.7532\times 10^{3}$	$3.8064\times 10^{3}$	$1.7707\times 10^{3}$	$2.3008\times 10^{3}$	$2.3113\times 10^{2}$
MGO	$1.8977\times 10^{4}$	$1.4618\times 10^{4}$	$2.0281\times 10^{4}$	$1.3320\times 10^{4}$	$2.1669\times 10^{3}$	$1.4905\times 10^{2}$
PO	$4.7931\times 10^{4}$	$3.1335\times 10^{4}$	$4.4915\times 10^{4}$	$4.0535\times 10^{4}$	$3.1861\times 10^{3}$	$4.2798\times 10^{2}$
BBO	$6.2347\times 10^{3}$	$4.4594\times 10^{3}$	$8.6640\times 10^{3}$	$7.3008\times 10^{3}$	$2.8120\times 10^{3}$	$3.1506\times 10^{2}$
DE	$5.1187\times 10^{4}$	$3.6061\times 10^{4}$	$7.0254\times 10^{3}$	$4.5995\times 10^{3}$	$2.0896\times 10^{3}$	$1.2257\times 10^{2}$
HGS	$4.9814\times 10^{4}$	$3.9098\times 10^{4}$	$1.6901\times 10^{4}$	$1.5673\times 10^{4}$	$2.7639\times 10^{3}$	$3.2363\times 10^{2}$
CPA	$8.3272\times 10^{3}$	$4.7165\times 10^{3}$	$2.9456\times 10^{3}$	$3.8388\times 10^{3}$	$2.7943\times 10^{3}$	$3.1053\times 10^{2}$
	**F16**		**F17**		**F18**	
**Algo.**	**Avg**	**Std**	**Avg**	**Std**	**Avg**	**Std**
CL-ECO	$1.8523\times 10^{3}$	$1.2993\times 10^{2}$	$1.8509\times 10^{3}$	$4.3668\times 10^{1}$	$1.9143\times 10^{3}$	$3.5801\times 10^{0}$
ECO	$1.9017\times 10^{3}$	$8.5150\times 10^{1}$	$1.3393\times 10^{5}$	$9.7375\times 10^{4}$	$6.3176\times 10^{3}$	$4.1862\times 10^{3}$
MGO	$1.8900\times 10^{3}$	$6.5934\times 10^{1}$	$3.1795\times 10^{5}$	$1.7934\times 10^{5}$	$1.3119\times 10^{4}$	$1.5376\times 10^{4}$
PO	$2.3027\times 10^{3}$	$2.2758\times 10^{2}$	$6.2835\times 10^{5}$	$6.0171\times 10^{5}$	$6.9727\times 10^{5}$	$5.3813\times 10^{5}$
BBO	$2.4568\times 10^{3}$	$2.8469\times 10^{2}$	$1.6261\times 10^{5}$	$1.0306\times 10^{5}$	$1.0223\times 10^{4}$	$7.6340\times 10^{3}$
DE	$1.8442\times 10^{3}$	$5.5836\times 10^{1}$	$3.0990\times 10^{5}$	$1.5814\times 10^{5}$	$8.1846\times 10^{3}$	$4.7661\times 10^{3}$
HGS	$2.2376\times 10^{3}$	$1.8271\times 10^{2}$	$2.8698\times 10^{5}$	$2.8627\times 10^{5}$	$1.3280\times 10^{4}$	$1.5377\times 10^{4}$
CPA	$2.2480\times 10^{3}$	$2.0772\times 10^{2}$	$1.1374\times 10^{5}$	$5.3790\times 10^{4}$	$4.7411\times 10^{3}$	$1.9190\times 10^{3}$
	**F19**		**F20**		**F21**	
**Algo.**	**Avg**	**Std**	**Avg**	**Std**	**Avg**	**Std**
CL-ECO	$2.1913\times 10^{3}$	$9.2027\times 10^{1}$	$2.3610\times 10^{3}$	$3.5005\times 10^{1}$	$2.6180\times 10^{3}$	$8.2506\times 10^{2}$
ECO	$2.2785\times 10^{3}$	$9.1827\times 10^{1}$	$2.3704\times 10^{3}$	$1.8209\times 10^{1}$	$2.9981\times 10^{3}$	$1.3088\times 10^{3}$
MGO	$2.2254\times 10^{3}$	$7.7426\times 10^{1}$	$2.3691\times 10^{3}$	$1.4021\times 10^{1}$	$2.8868\times 10^{3}$	$1.2935\times 10^{3}$
PO	$2.5498\times 10^{3}$	$1.6197\times 10^{2}$	$2.4712\times 10^{3}$	$5.7331\times 10^{1}$	$3.5889\times 10^{3}$	$2.0994\times 10^{3}$
BBO	$2.6834\times 10^{3}$	$2.1113\times 10^{2}$	$2.4703\times 10^{3}$	$3.9095\times 10^{1}$	$5.3078\times 10^{3}$	$2.2245\times 10^{3}$
DE	$2.1454\times 10^{3}$	$6.3978\times 10^{1}$	$2.4093\times 10^{3}$	$8.9045\times 10^{0}$	$3.7314\times 10^{3}$	$1.7251\times 10^{3}$
HGS	$2.5478\times 10^{3}$	$1.9454\times 10^{2}$	$2.4186\times 10^{3}$	$2.8809\times 10^{1}$	$5.1291\times 10^{3}$	$1.3596\times 10^{3}$
CPA	$2.4756\times 10^{3}$	$2.2246\times 10^{2}$	$2.3900\times 10^{3}$	$8.0661\times 10^{1}$	$3.6050\times 10^{3}$	$1.7662\times 10^{3}$
	**F22**		**F23**		**F24**	
**Algo.**	**Avg**	**Std**	**Avg**	**Std**	**Avg**	**Std**
CL-ECO	$2.7157\times 10^{3}$	$1.8044\times 10^{1}$	$2.9441\times 10^{3}$	$4.1768\times 10^{1}$	$2.8859\times 10^{3}$	$1.9808\times 10^{0}$
ECO	$2.7151\times 10^{3}$	$1.6414\times 10^{1}$	$2.8828\times 10^{3}$	$1.6664\times 10^{1}$	$2.8889\times 10^{3}$	$1.0204\times 10^{1}$
MGO	$2.7232\times 10^{3}$	$1.5514\times 10^{1}$	$2.8962\times 10^{3}$	$1.8100\times 10^{1}$	$2.8877\times 10^{3}$	$1.1836\times 10^{1}$
PO	$2.9531\times 10^{3}$	$6.9655\times 10^{1}$	$3.1198\times 10^{3}$	$6.3845\times 10^{1}$	$2.9338\times 10^{3}$	$3.2522\times 10^{1}$
BBO	$3.2887\times 10^{3}$	$1.6665\times 10^{2}$	$3.3369\times 10^{3}$	$1.2719\times 10^{2}$	$2.8797\times 10^{3}$	$5.5324\times 10^{0}$
DE	$2.7540\times 10^{3}$	$9.5522\times 10^{0}$	$2.9569\times 10^{3}$	$1.2830\times 10^{1}$	$2.8872\times 10^{3}$	$8.1962\times 10^{-1}$
HGS	$2.7637\times 10^{3}$	$2.1612\times 10^{1}$	$3.0398\times 10^{3}$	$6.5270\times 10^{1}$	$2.8878\times 10^{3}$	$7.4414\times 10^{0}$
CPA	$2.7624\times 10^{3}$	$3.0974\times 10^{1}$	$3.0889\times 10^{3}$	$8.0677\times 10^{1}$	$2.8981\times 10^{3}$	$1.7622\times 10^{1}$
	**F25**		**F26**		**F27**	
**Algo.**	**Avg**	**Std**	**Avg**	**Std**	**Avg**	**Std**
CL-ECO	$3.7553\times 10^{3}$	$7.5610\times 10^{2}$	$3.2070\times 10^{3}$	$1.5429\times 10^{1}$	$3.1299\times 10^{3}$	$5.6329\times 10^{1}$
ECO	$3.8213\times 10^{3}$	$8.5085\times 10^{2}$	$3.2119\times 10^{3}$	$1.1218\times 10^{1}$	$3.1558\times 10^{3}$	$5.0975\times 10^{1}$
MGO	$4.1601\times 10^{3}$	$4.2064\times 10^{2}$	$3.2120\times 10^{3}$	$6.5345\times 10^{0}$	$3.2291\times 10^{3}$	$1.6278\times 10^{1}$
PO	$6.6578\times 10^{3}$	$1.3520\times 10^{3}$	$3.3195\times 10^{3}$	$4.6267\times 10^{1}$	$3.3066\times 10^{3}$	$2.3959\times 10^{1}$
BBO	$6.1542\times 10^{3}$	$2.4901\times 10^{3}$	$3.3781\times 10^{3}$	$3.6078\times 10^{2}$	$3.1425\times 10^{3}$	$5.3970\times 10^{1}$
DE	$4.6762\times 10^{3}$	$9.9029\times 10^{1}$	$3.2067\times 10^{3}$	$3.7893\times 10^{0}$	$3.1896\times 10^{3}$	$5.9495\times 10^{1}$
HGS	$4.8239\times 10^{3}$	$5.9969\times 10^{2}$	$3.2243\times 10^{3}$	$1.6836\times 10^{1}$	$3.2195\times 10^{3}$	$4.1353\times 10^{1}$
CPA	$4.3087\times 10^{3}$	$1.3789\times 10^{3}$	$3.2380\times 10^{3}$	$2.1069\times 10^{1}$	$3.1464\times 10^{3}$	$4.5445\times 10^{1}$
	**F28**		**F29**			
**Algo.**	**Avg**	**Std**	**Avg**	**Std**		
CL-ECO	$3.4238\times 10^{3}$	$7.4625\times 10^{1}$	$5.1519\times 10^{3}$	$1.8974\times 10^{2}$		
ECO	$3.5855\times 10^{3}$	$1.3411\times 10^{2}$	$1.3599\times 10^{4}$	$4.3022\times 10^{3}$		
MGO	$3.6213\times 10^{3}$	$8.1123\times 10^{1}$	$8.6405\times 10^{4}$	$8.7166\times 10^{4}$		
PO	$4.5898\times 10^{3}$	$2.5888\times 10^{2}$	$6.0770\times 10^{6}$	$5.0656\times 10^{6}$		
BBO	$3.9869\times 10^{3}$	$2.7247\times 10^{2}$	$5.5105\times 10^{3}$	$1.8567\times 10^{3}$		
DE	$3.4987\times 10^{3}$	$7.7710\times 10^{1}$	$1.2277\times 10^{4}$	$5.1841\times 10^{3}$		
HGS	$3.7420\times 10^{3}$	$1.6531\times 10^{2}$	$8.8430\times 10^{4}$	$1.1080\times 10^{5}$		
CPA	$3.6656\times 10^{3}$	$1.9220\times 10^{2}$	$1.3805\times 10^{4}$	$7.3601\times 10^{3}$		
**Overall Rank**						
**Algo.**	**RANK**	**+/=/−**	**AVG**			
CL-ECO	1	∼	1.5862			
ECO	2	19/8/2	3.3103			
MGO	4	19/8/2	4.1724			
PO	8	29/0/0	7.5172			
BBO	6	24/3/2	5.4483			
DE	3	22/3/4	4.0			
HGS	7	28/1/0	5.7586			
CPA	5	27/0/2	4.2069			

**Table 3:** The *p*-values of the CL-ECO vs. other algorithms on CEC2017.

Fun	ECO	MGO	PO	BBO	DE	HGS	CPA
F1	$1.73\times 10^{-6}$	$1.73\times 10^{-6}$	$1.73\times 10^{-6}$	$1.73\times 10^{-6}$	$1.73\times 10^{-6}$	$1.73\times 10^{-6}$	$1.73\times 10^{-6}$
F2	$1.92\times 10^{-6}$	$1.73\times 10^{-6}$	$1.73\times 10^{-6}$	$1.73\times 10^{-6}$	$1.73\times 10^{-6}$	$1.73\times 10^{-6}$	$1.73\times 10^{-6}$
F3	$2.13\times 10^{-6}$	$1.73\times 10^{-6}$	$1.73\times 10^{-6}$	$1.64\times 10^{-5}$	$1.73\times 10^{-6}$	$2.60\times 10^{-6}$	$1.73\times 10^{-6}$
F4	$6.04\times 10^{-3}$	$2.06\times 10^{-1}$	$1.73\times 10^{-6}$	$1.73\times 10^{-6}$	$1.73\times 10^{-6}$	$1.73\times 10^{-6}$	$1.92\times 10^{-6}$
F5	$1.73\times 10^{-6}$	$8.59\times 10^{-2}$	$1.73\times 10^{-6}$	$1.73\times 10^{-6}$	$1.73\times 10^{-6}$	$1.73\times 10^{-6}$	$4.58\times 10^{-5}$
F6	$3.18\times 10^{-6}$	$1.48\times 10^{-2}$	$1.73\times 10^{-6}$	$1.73\times 10^{-6}$	$1.73\times 10^{-6}$	$1.73\times 10^{-6}$	$2.13\times 10^{-6}$
F7	$2.54\times 10^{-1}$	$8.22\times 10^{-3}$	$1.73\times 10^{-6}$	$1.73\times 10^{-6}$	$1.73\times 10^{-6}$	$1.73\times 10^{-6}$	$2.60\times 10^{-6}$
F8	$2.41\times 10^{-3}$	$1.11\times 10^{-2}$	$1.73\times 10^{-6}$	$1.73\times 10^{-6}$	$1.73\times 10^{-6}$	$1.73\times 10^{-6}$	$1.73\times 10^{-6}$
F9	$3.88\times 10^{-6}$	$1.73\times 10^{-6}$	$1.73\times 10^{-6}$	$1.73\times 10^{-6}$	$1.73\times 10^{-6}$	$1.92\times 10^{-6}$	$1.97\times 10^{-5}$
F10	$1.73\times 10^{-6}$	$1.92\times 10^{-6}$	$1.73\times 10^{-6}$	$1.92\times 10^{-6}$	$6.98\times 10^{-6}$	$1.73\times 10^{-6}$	$3.52\times 10^{-6}$
F11	$1.73\times 10^{-6}$	$1.73\times 10^{-6}$	$1.73\times 10^{-6}$	$1.92\times 10^{-6}$	$1.73\times 10^{-6}$	$1.73\times 10^{-6}$	$1.73\times 10^{-6}$
F12	$1.73\times 10^{-6}$	$1.73\times 10^{-6}$	$1.73\times 10^{-6}$	$1.73\times 10^{-6}$	$1.73\times 10^{-6}$	$1.73\times 10^{-6}$	$1.73\times 10^{-6}$
F13	$1.73\times 10^{-6}$	$1.73\times 10^{-6}$	$1.73\times 10^{-6}$	$1.73\times 10^{-6}$	$1.73\times 10^{-6}$	$1.73\times 10^{-6}$	$1.73\times 10^{-6}$
F14	$1.73\times 10^{-6}$	$1.73\times 10^{-6}$	$1.73\times 10^{-6}$	$1.73\times 10^{-6}$	$1.73\times 10^{-6}$	$1.73\times 10^{-6}$	$1.73\times 10^{-6}$
F15	$2.06\times 10^{-1}$	$3.60\times 10^{-1}$	$2.35\times 10^{-6}$	$2.35\times 10^{-6}$	$1.25\times 10^{-2}$	$1.73\times 10^{-6}$	$2.88\times 10^{-6}$
F16	$7.19\times 10^{-2}$	$1.92\times 10^{-1}$	$3.18\times 10^{-6}$	$1.73\times 10^{-6}$	$8.61\times 10^{-1}$	$3.88\times 10^{-6}$	$2.88\times 10^{-6}$
F17	$1.73\times 10^{-6}$	$1.73\times 10^{-6}$	$1.73\times 10^{-6}$	$1.73\times 10^{-6}$	$1.73\times 10^{-6}$	$1.73\times 10^{-6}$	$1.73\times 10^{-6}$
F18	$1.73\times 10^{-6}$	$1.73\times 10^{-6}$	$1.73\times 10^{-6}$	$1.73\times 10^{-6}$	$1.73\times 10^{-6}$	$1.73\times 10^{-6}$	$1.73\times 10^{-6}$
F19	$1.29\times 10^{-3}$	$1.53\times 10^{-1}$	$1.73\times 10^{-6}$	$1.73\times 10^{-6}$	$4.28\times 10^{-2}$	$2.35\times 10^{-6}$	$1.64\times 10^{-5}$
F20	$3.09\times 10^{-1}$	$3.82\times 10^{-1}$	$7.69\times 10^{-6}$	$1.73\times 10^{-6}$	$2.13\times 10^{-6}$	$5.75\times 10^{-6}$	$2.18\times 10^{-2}$
F21	$4.99\times 10^{-3}$	$1.20\times 10^{-3}$	$6.64\times 10^{-4}$	$1.12\times 10^{-4}$	$2.77\times 10^{-3}$	$4.29\times 10^{-6}$	$8.15\times 10^{-4}$
F22	$7.81\times 10^{-1}$	$1.31\times 10^{-1}$	$1.73\times 10^{-6}$	$1.73\times 10^{-6}$	$1.92\times 10^{-6}$	$1.92\times 10^{-6}$	$1.73\times 10^{-6}$
F23	$4.29\times 10^{-6}$	$3.18\times 10^{-6}$	$1.92\times 10^{-6}$	$1.73\times 10^{-6}$	$7.52\times 10^{-2}$	$2.88\times 10^{-6}$	$1.92\times 10^{-6}$
F24	$5.45\times 10^{-2}$	$5.31\times 10^{-5}$	$1.73\times 10^{-6}$	$2.41\times 10^{-4}$	$1.38\times 10^{-3}$	$1.20\times 10^{-1}$	$3.32\times 10^{-4}$
F25	$7.50\times 10^{-1}$	$2.30\times 10^{-2}$	$2.60\times 10^{-6}$	$2.05\times 10^{-4}$	$1.73\times 10^{-6}$	$4.86\times 10^{-5}$	$4.07\times 10^{-2}$
F26	$2.62\times 10^{-1}$	$9.37\times 10^{-2}$	$1.73\times 10^{-6}$	$7.66\times 10^{-1}$	$7.81\times 10^{-1}$	$7.16\times 10^{-4}$	$3.52\times 10^{-6}$
F27	$1.32\times 10^{-2}$	$1.02\times 10^{-5}$	$1.73\times 10^{-6}$	$7.94\times 10^{-2}$	$2.40\times 10^{-3}$	$1.36\times 10^{-5}$	$4.07\times 10^{-2}$
F28	$7.69\times 10^{-6}$	$6.98\times 10^{-6}$	$1.73\times 10^{-6}$	$1.92\times 10^{-6}$	$2.96\times 10^{-3}$	$1.73\times 10^{-6}$	$1.49\times 10^{-5}$
F29	$1.73\times 10^{-6}$	$1.73\times 10^{-6}$	$1.73\times 10^{-6}$	$3.93\times 10^{-1}$	$1.73\times 10^{-6}$	$1.73\times 10^{-6}$	$1.73\times 10^{-6}$

**Table 4:** Experimental results of all algorithms on the production optimization problem.

Algorithm	Mean (USD)	Std	Best (USD)	Worst (USD)
CL-ECO	$9.842\times 10^{8}$	$1.273\times 10^{7}$	$1.001\times 10^{9}$	$9.614\times 10^{8}$
ECO	$8.745\times 10^{8}$	$3.112\times 10^{7}$	$9.127\times 10^{8}$	$8.285\times 10^{8}$
DE	$9.136\times 10^{8}$	$2.453\times 10^{7}$	$9.521\times 10^{8}$	$8.748\times 10^{8}$
MGO	$8.387\times 10^{8}$	$3.771\times 10^{7}$	$8.806\times 10^{8}$	$7.892\times 10^{8}$
PO	$8.043\times 10^{8}$	$4.337\times 10^{7}$	$8.491\times 10^{8}$	$7.498\times 10^{8}$
HGS	$8.521\times 10^{8}$	$3.864\times 10^{7}$	$8.932\times 10^{8}$	$8.075\times 10^{8}$
BBO	$8.923\times 10^{8}$	$2.981\times 10^{7}$	$9.362\times 10^{8}$	$8.456\times 10^{8}$
CPA	$8.612\times 10^{8}$	$3.428\times 10^{7}$	$8.991\times 10^{8}$	$8.137\times 10^{8}$

## Data Availability

The numerical and experimental data used to support the findings of this study are included within the article.

## Abbreviations

The following abbreviations are used in this manuscript.

CL-ECO	Comprehensive Learning-Enhanced Educational Competition Optimizer
ECO	Educational Competition Optimizer
CL	Comprehensive Learning
NPV	Net Present Value
BHP	Bottom-Hole Pressure
RPO	Reservoir Production Optimization
CEC	Congress on Evolutionary Computation
EA	Evolutionary Algorithm
SI	Swarm Intelligence
GA	Genetic Algorithm
DE	Differential Evolution
PSO	Particle Swarm Optimization
ACO	Ant Colony Optimization
NFL	No Free Lunch
